# Eosinophilic Annular Erythema: A New Entity of Eosinophilic Dermatosis

**DOI:** 10.7759/cureus.22657

**Published:** 2022-02-27

**Authors:** Madiha Eljazouly, Fatimazahra Chahboun, Maha Alj, Kenza Oqbani, Soumiya Chiheb

**Affiliations:** 1 Dermatology Unit, Cheikh Khalifa International University Hospital, Mohammed VI University of Health Sciences, Casablanca, MAR; 2 Pathology, Cheikh Khalifa International University Hospital, Mohammed VI University of Health Sciences, Casablanca, MAR

**Keywords:** steroids, synthetic antimalarials, annular erythema, eosinophilic cellulitis, wells syndrome, eosinophilic dermatosis

## Abstract

Eosinophilic annular erythema (EAE) is a rare dermatosis. Its relationship with Wells syndrome (WS) is debated. We report a case treated with hydroxychloroquine.

A 31-year-old patient presented with a mildly pruritic rash that had been evolving by flares for two weeks. Clinical examination revealed inflammatory erythematous-annular plaques on the trunk and limbs. The blood count was normal. Skin histology showed an eosinophilic-rich inflammatory infiltrate. After local steroid treatment, the patient was treated with oral steroids with a momentary improvement. The course with new relapses is treated by synthetic antimalarial drugs with the complete disappearance of the lesions at a six-month follow-up.

Although some authors consider EAE to be a variant of WS, we believe that there are subtle differences that differentiate them despite their clinical similarity.

## Introduction

Eosinophilic annular erythema (EAE) is an annular dermatosis recently described raising different questions. It initially raised a nosological problem that discusses its belonging to the eosinophilic dermatosis and its relationship with the Wells syndrome (WS). Currently, the debate also concerns therapeutic management. EAE is characterized clinically by recurrent urticarial and annular lesions with a tendency to centrifugal extension and central healing associated with dermal infiltration of eosinophils and, in some cases, blood eosinophilia. The disease course is often chronic, recurrent, and relapsing [1]. Responses to treatment are variable but are generally best with systemic steroids and antimalarials [2]. We report a new case treated with hydroxychloroquine.

## Case presentation

A 31-year-old man, with no previous medical history, was admitted to the emergency department for inflammatory and itchy skin lesions involving the trunk, limbs, and gluteal region. There was no medical history of drug intake. The history of the disease goes back two weeks before his admission with the installation of papular and urticarial lesions of the trunk and limbs. The patient was treated with local Benzyl Benzoate and antihistaminic without improvement.

**Figure 1:**
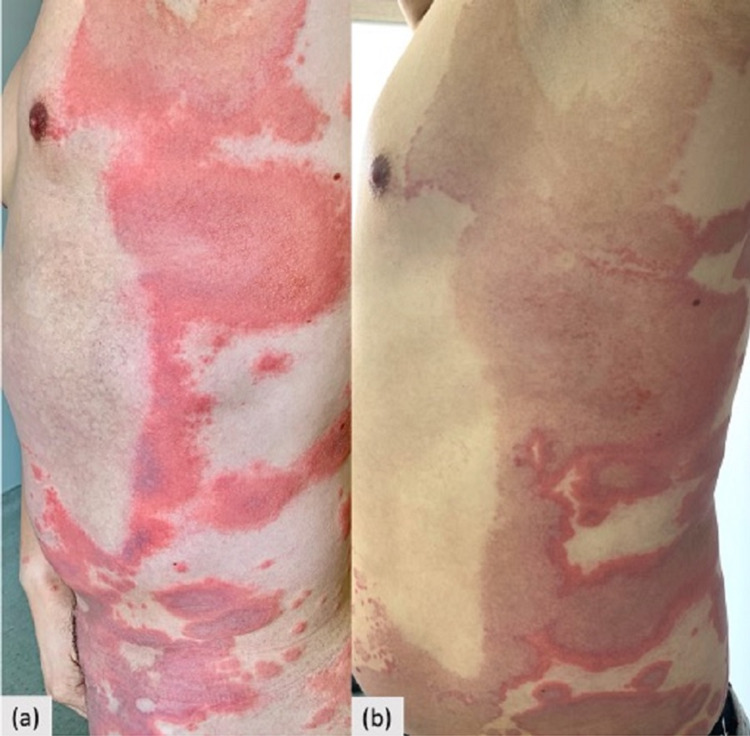
(a) Erythematous annular plaques on the trunk. (b) Evolution after local steroids with central hyperpigmentation.

On admission, the physical examination revealed annular erythematous and partially serpiginous plaques with central hyperpigmentation and edematous borders located on the trunk, the back, the limbs, and the gluteal region (Figures 1a, 1b). Physical examination was otherwise normal.

Several diagnoses were initially discussed, including toxidermia, giant urticaria, sweet syndrome, WS, and subacute lupus.

**Figure 2:**
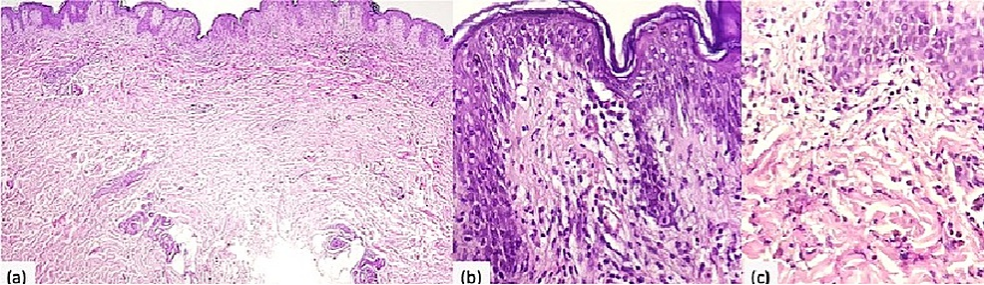
Perivascular and interstitial infiltration with abundant eosinophils; flame figures were not found. (a) Hematoxylin and eosin (HE) x4 and (b, c) HE x40.

Routine laboratory tests did not disclose any abnormality and included complete blood count, erythrocyte sedimentation, rate C-reactive protein, renal and liver function. Screening antibodies for antinuclear, anti-DNA, anti-SS-A/Ro, and anti-SS-B/La, were all negative. The serology for hepatitis B and C, HIV, Epstein bar virus, cytomegalovirus, and parvovirus B19 were also negative. Electrophoretogram and serum immune electrophoretogram were normal. The urea breath test was positive. Radiological investigations (chest x-ray and abdominal ultrasonography) were normal. The skin biopsy showed an eosinophilic-rich inflammatory infiltrate in the deep layers of the epidermis and with a perivascular arrangement in the dermis (Figures 2a-2c).

**Figure 3:**
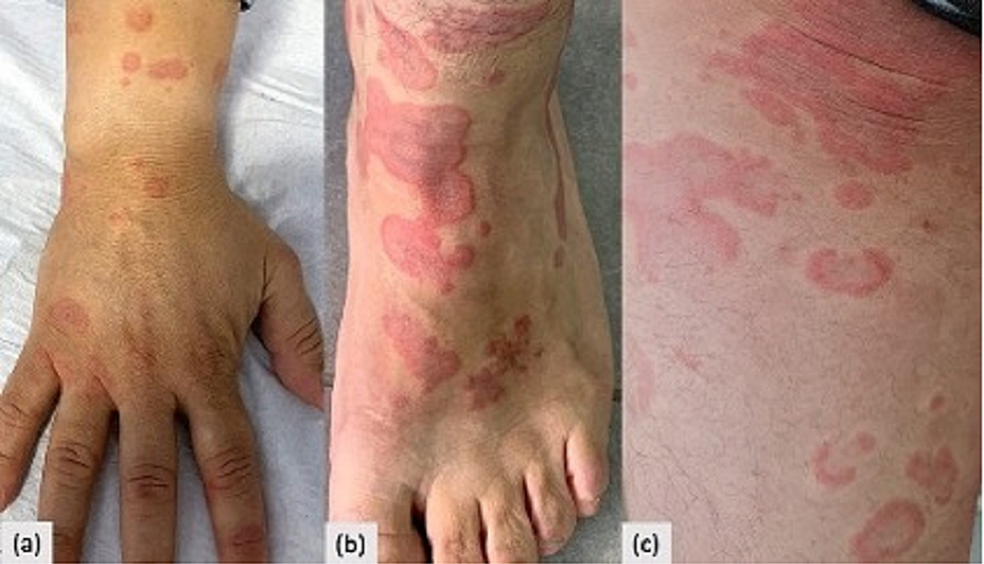
Relapse of polycyclic annular erythematous lesions on the hands (a), back of the feet (b), and thigh (c).

The patient was initially treated with local steroids and antihistaminic with temporary improvement. Oral corticosteroids were subsequently started with a momentary improvement and new lesions continued to flare (Figures 3a-3c). Therefore, the patient, with the clinical and histopathological orientation of EAE, was treated with hydroxychloroquine (6.5mg/kg/day). Six months later there was no recurrence, with complete clearance of the plaques.

## Discussion

EAE was first described by Peterson and Jarratt in 1981 included only pediatric patients. Thus, it was initially named annular erythema of infancy characterized by cyclic and erythematous lesions affecting predominantly the trunk and proximal areas of the extremities. Eruption may persist for several weeks or months [3]. In 2000, Kahofer et al. reported the first case in an adult. Its classification as a new clinical entity, a variant of Wells' syndrome, or a subtype of annular erythemas remains debated. Both, belong to the spectrum of eosinophilic dermatosis [4].

Another diagnosis is subject to controversy, the overlap syndrome (sweet syndrome and WS) or (sweet syndrome associated with Infiltration of eosinophils), which is distinguished clinically by highly inflammatory necrotic ring lesions with fever and histologically by a neutrophil- and eosinophil-rich infiltrate without vasculitis [5].

Our observation illustrates the clinical feature of EAE described by the authors. EAE presents several clinical and histological differences. It is a recurrent dermatosis that manifests as erythematous-papular ring lesions with central healing. This figurative pattern has also been reported in WS. However, the prodromal features and the blood hypereosinophilia usually found in WS were missing. Histologically, the EAE is characterized by a superficial perivascular and interstitial mixed infiltrate with prominent eosinophils. Although some authors reported that the histological appearance may be similar to Wells Syndrome, especially as the biopsy is performed late in a well-developed lesion. It is showed superficial and deep infiltrate, with abundant eosinophils and flame figures. This underlines that EAE is a particular clinical variant of WS [1,6].

In addition to the WS, several differential diagnoses have been proposed including erythema chronicum migrans, erythema annular centrifugum, erythema gyratum repens, cutaneous annular sarcoidosis, and Granuloma annulare in localized forms [7,8].

The pathogenesis of EAE remains unclear, but it is suggested that it may be a hypersensitivity reaction to an unidentified antigen. Thus, several hypotheses have been put forward in this sense, involving the role of IL-5 in the recruitment of eosinophils in response to certain stimuli such as insect bites. Another hypothesis concerns the presence of a receptor for IL-2 (CD25) on the surface of eosinophils, which causes or participates in their granulation [9,10]. Other published cases suggest the association of EAE with several chronic diseases such as autoimmune thyroid disease, diabetes mellitus, chronic borreliosis, autoimmune hepatitis, hepatitis C infection, hematological disorders, systemic lupus erythematosus, and rheumatoid arthritis, as well as chronic Helicobacter pylori gastritis, as in our patient [7]. Although suspected to be a hypersensitivity reaction, association with internal malignancy has been reported, including renal cell carcinoma, metastatic adenocarcinoma of the prostate, thymoma, which completely resolved after thymectomy, and breast cancer [7,10].

The treatment is mainly symptomatic and the mechanism of action is not completely understood. Eosinophils are considered to be crucial cells in the development of EAE. Thus systemic steroids and antimalarials are the most commonly used treatment option. Indeed, the efficacy of antimalarials has been suggested due to their ability to inhibit eosinophilotaxis and release proinflammatory cytokines [4]. Most reports in the literature note the use of antimalarials as the first line for EAE, response to hydroxychloroquine is typically prompt and usually observed within 2-4 weeks. However, some authors have reported a delayed response to chloroquine and have decided to prolong the treatment to avoid relapse [2]. Nevertheless, many cases are resistant to treatment with antimalarials suggestive that systemic corticosteroids remain the treatment of choice in EAE. In our patient, the relay with hydroxychloroquine was effective. He also received treatment for Helicobacter pylori. Dapsone treatment has been reported in several studies with patients showing a good response. It has been found to inhibit the eosinophil peroxidase and subsequently block the mast cell degranulation and activation [11]. Other treatment options have also been tested in isolated cases such as indometacin, nicotinamide, methotrexate, cyclosporine A, and mycophenolate mofetil. There was also a case report of a child who achieved complete resolution of EAE after phototherapy UVB. In a recent study, Magdalena et al. had tried mepolizumab in the treatment of EAE with a good outcome. It is a humanized monoclonal antibody that targets IL-5, a cytokine crucial for the development and tissue migration of eosinophils. Therefore, targeting cytokines crucial to eosinophil function could be a new direction in the treatment of EAE, but further studies are needed to provide additional evidence [12].

## Conclusions

In conclusion, EAE is considered a variant of WS although there are or subsist several clinical and essentially histological differences between these two entities. It is characterized by a chronic course, resistance to treatment, and a high relapse rate. In addition, the diagnosis and the evaluation of this condition need repeated monitoring with clinical, radiological, and laboratory assessments.

Thus, the development or study of the functional mechanisms of eosinophils could in the future elucidate the pathogenesis of this eosinophilic dermatosis for better therapeutic management.
